# Electrochemical Sensing of Glucose Using Glucose Oxidase/PEDOT:4-Sulfocalix [4]arene/MXene Composite Modified Electrode

**DOI:** 10.3390/mi13020304

**Published:** 2022-02-16

**Authors:** Preethika Murugan, Jayshree Annamalai, Raji Atchudan, Mani Govindasamy, Deepak Nallaswamy, Dhanraj Ganapathy, Anatoly Reshetilov, Ashok K. Sundramoorthy

**Affiliations:** 1 Department of Chemistry, SRM Institute of Science and Technology, Kattankulathur 603203, Tamil Nadu, India; preethikam95@gmail.com; 2 Department of Biotechnology, SRM Institute of Science and Technology, Kattankulathur 603203, Tamil Nadu, India; jayshreeannamalai@yahoo.com; 3 School of Chemical Engineering, Yeungnam University, Gyeongsan 38541, Korea; atchudanr@yu.ac.kr; 4 Department of Materials Engineering, Ming-Chi University of Technology, New Taipei City 243, Taiwan; govindasamy420700@gmail.com; 5 Department of Prosthodontics, Saveetha Dental College and Hospitals, Saveetha Institute of Medical and Technical Sciences, Poonamallee High Road, Velappanchavadi, Chennai 600077, Tamil Nadu, India; dir.acad.sac@saveetha.com (D.N.); dhanraj@saveetha.com (D.G.); 6 G.K. Skryabin Institute of Biochemistry and Physiology of Microorganisms, Pushchino Centre for Biological Research, Russian Academy of Sciences, 142290 Pushchino, Russia; anatol@ibpm.pushchino.ru

**Keywords:** PEDOT, 4-sulfocalix [4]arene, MXene, glucose oxidase, electrochemical sensor

## Abstract

Glucose is one of the most important monosaccharides found in the food, as a part of more complex structures, which is a primary energy source for the brain and body. Thus, the monitoring of glucose concentration is more important in food and biological samples in order to maintain a healthy lifestyle. Herein, an electrochemical glucose biosensor was fabricated by immobilization of glucose oxidase (GOX) onto poly(3,4-ethylenedioxythiophene):4-sulfocalix [4]arene (PEDOT:SCX)/MXene modified electrode. For this purpose, firstly, PEDOT was synthesized in the presence of SCX (counterion) by the chemical oxidative method. Secondly, MXene (a 2D layered material) was synthesized by using a high-temperature furnace under a nitrogen atmosphere. After that, PEDOT:SCX/MXene (1:1) dispersion was prepared by ultrasonication which was later utilized to prepare PEDOT:SCX/MXene hybrid film. A successful formation of PEDOT:SCX/MXene film was confirmed by HR-SEM, Fourier transform infrared (FT-IR), and Raman spectroscopies. Due to the biocompatibility nature, successful immobilization of GOX was carried out onto chitosan modified PEDOT:SCX/MXene/GCE. Moreover, the electrochemical properties of PEDOT:SCX/MXene/GOX/GCE was studied through cyclic voltammetry and amperometry methods. Interestingly, a stable redox peak of FAD-GOX was observed at a formal potential of –0.435 V on PEDOT:SCX/MXene/GOX/GCE which indicated a direct electron transfer between the enzyme and the electrode surface. PEDOT:SCX/MXene/GOX/GCE also exhibited a linear response against glucose concentrations in the linear range from 0.5 to 8 mM. The effect of pH, sensors reproducibility, and repeatability of the PEDOT:SCX/MXene/GOX/GCE sensor were studied. Finally, this new biosensor was successfully applied to detect glucose in commercial fruit juice sample with satisfactory recovery.

## 1. Introduction

In the field of medical diagnosis and personal healthcare, diabetes mellitus is one of the leading causes of death and disability of humans all around the globe [1]. Thus, it is essential to monitor the glucose levels accurately in biological samples so that few complications such as heart disease, kidney failure, and blindness can be treated at their initial stages [2]. Similarly, glucose has been involved in some of the most essential processes and metabolism, for example, photosynthesis and respiration. Glucose has been also used as a substrate for yeast in the process of fermentation and also as a flavour enhancer in the food manufacturing and packaging industries [3]. So, the detection of glucose in blood and food samples must be rapid, accurate, and cheap [4,5,6]. Generally, the glucose concentration can be measured by various methods such as electrochemical [7,8], photometry [9,10], chemiluminescence [11,12], electro-chemiluminescence [13,14], flow injection analysis [15,16], chromatography [17], UV-Vis spectroscopy [18], etc. 

Among these methods, electrochemical sensors can be used to detect glucose by either enzymatic or non-enzymatic methods [19]. Generally, non-enzymatic sensors had shown some limitations such as poor selectivity, low sensitivity in physiological pH, quick surface poisoning by the intermediate adsorption and loss of sensor activity [20]. To avoid these problems, electrochemical enzymatic biosensors have been developed for the accurate detection of glucose. Usually, glucose oxidase (GOX) and glucose dehydrogenase enzymes are commonly utilized to fabricate glucose biosensors [21]. Herein, we have fabricated a biosensor using GOX, a flavin enzyme, with a molar weight of about 150–180 kDa [22]. GOX is a redox-active protein whose active site is a flavin adenine dinucleotide (FAD) which is deeply embedded in the protein shell [23]. Clark and Lyons [24] were the ones who first projected the initial concept of glucose biosensor fabricated with GOX which has been broadly studied to improve their performance and applications in biomedical and clinical trials [25]. When GOX was directly adsorbed on the surface of the electrode, the bioactivity of GOX was also affected due to denaturation [26]. Numerous approaches have been reported for effective enzyme immobilization of GOX on the electrode surface through cross-linking, chemical polymerization, sol-gel encapsulation, and surface adsorption strategies [27]. The above electrode modification methods were developed by using a variety of materials such as multi-walled carbon nanotube/Nafion [28], self-assembled monolayers [29], conducting polymers like polyaniline [30], polythiophene [31], polypyrrole [32], etc. Ultimately, the direct electron transfer between the redox groups of enzymes and conductive nanomaterials were important for the successful preparation of electrochemical sensor devices [33].

Recently, a variety of 2D layered materials have been synthesized for various applications [34,35,36]. Specifically, researchers have concentrated on the exclusive physical and chemical properties of a single layer of graphene [37,38,39] and also on the other non-graphene layered materials [40]. MXene (M_n+1_X_n_T_x_), a transition metal carbides-based layered material, was first reported by Yury Gogotsi in 2011 [41]. In MXene structure, M denotes transition metal (M), X denotes the carbon or nitrogen, and T is the surface functional group, such as F^−^, O^−^, OH^−^ and Cl^−^ [38,42,43,44]. Layered MXene sheets were derived after the removal of an element from its MAX phase by an etching process [45,46,47]. MXene had exhibited hydrophilic properties and metallic nature due to the presence of terminal functional groups [48,49]. MXene had been used in various electrochemical applications such as sensors [50,51,52,53], energy storage systems [54], oxygen evolution reaction, and hydrogen evolution reaction [55]. Generally, MXene (Ti_3_C_2_T_x_) synthesized as a mixed-phase was found to have poor dispersibility in water. To increase the dispersibility of MXene in an aqueous solvent, we have used water soluble poly 3,4-ethylene dioxythiophene (PEDOT) as a dispersant in this work.

Similarly, various conducting polymers (examples: polyacetylene, polypyrrole, polyaniline, PEDOT, and polythiophene) have been used in the designing of advanced bioelectrodes for catalysis and sensors [56]. Compared to the traditional methods, the metal nanoparticles have been used to immobilize the GOX enzyme to realize the bioelectrocatalytic activity [57]. However, the non-enzymatic electrochemical sensors performance depended on the nature of metal nanoparticles for glucose detection [58,59]. In the case of polymers, they may become a conducting polymer when an electron was removed (cation) or after the addition of an electron (anion) [60]. These anion and cation of polymer acted as the “charge carriers” under the effect of an electric field, therefore the conductivity of the polymer is increased [61]. Doping is an efficient way to synthesise conducting polymers with attractive properties for various applications [62] and it allows the electrons to flow by the formation of a conduction band [63]. PEDOT is one of such conducting polymers which can be synthesized by wet-chemical oxidation methods by using iron catalyst [64], liquid/liquid interfacial [65], electrochemical [66], and vapour phase polymerization protocols [67]. Generally, PEDOT was derived from polythiophene with many advantages such as good thermal stability, low oxidation potential, high film-forming ability, good transparency, and high electrical conductivity [68]. It can be found that one positive charge density per every three EDOT monomer units which were in the equilibrium state [69]. Thus, it is necessary to have one charge balancing anion per three EDOT units of the polymer chain. So far, different types of anions were used for PEDOT doping such as ClO_4_^−^, heparin, polystyrene sulfonate (PSS), etc. [70]. 

In this study, we have polymerized EDOT monomers with 4-sulfocalix [4]arene (SCX) as a dopant. SCX is a cup-shaped anionic molecule with an amphiphilic nature due to the presence of hydrophilic groups (SO_3_^−^) and hydrophobic groups (benzene rings) [71,72]. Firstly, PEDOT was obtained by the chemical oxidative method using iron chloride as the oxidant in the presence of SCX counter ions. Secondly, the PEDOT:SCX solution was used to disperse MXene by ultra-sonication to prepare PEDOT:SCX/MXene film. The Fourier transform infrared (FT-IR) and Raman spectroscopies were used to investigate the functional groups, metal-oxygen vibrations and crystalline defects present on the surface. The surface morphology of PEDOT:SCX/MXene was studied by high resolution-scanning electron microscopy (HR-SEM). To investigate the potential application of the nanocomposite, PEDOT:SCX/MXene film was prepared and used to immobilize GOX using chitosan as a binder [73]. 

The stability of the enzyme on the electrode surface was more important to observe the enhanced electrocatalytic activity with better operational stability [74,75,76]. As-prepared PEDOT:SCX/MXene/GOX was applied for the electrochemical detection of glucose. It exhibited good electrochemical activity through the redox peak of FAD at the formal potential of −0.435 V [77]. In addition, the effect of pH and scan rate of the biosensor were investigated which showed high bio-activity in the physiological pH. Interestingly, PEDOT:SCX/MXene/GOX coated GCE responded linearly with the additions of glucose from 0.5–8 mM, and the limit of detection (LOD) was found to be 22.5 µM. Moreover, detection of glucose concentration was demonstrated in a fruit juice sample by using PEDOT:SCX/MXene/GOX coated GCE with satisfactory recovery.

## 2. Experimental

### 2.1. Chemicals and Reagents

The GOX was obtained from Aspergillus Niger, 3,4-ethylene dioxythiophene (EDOT), 4-sulfocalix [4]arene (SCX) and chitosan (from crab shells, minimum 85% deacetylation) were purchased from Sigma-Aldrich, Bangalore, India. All the chemicals were of analytical grade and used without any further purifications. Iron chloride (FeCl_3_), titanium metal powder (325 mesh), aluminium metal powder (325 mesh), graphite nanopowder (400 nm), oxalic acid (OA), ascorbic acid (AA), uric acid (UA), L-alanine (L-ala), L–tyrosine (L-try) and hydrofluoric acid (40% HF) were purchased from SRL India. Phosphate buffer solution (0.1 M, PBS) (pH 7.4) was prepared using sodium monohydrogen phosphate (Na_2_HPO_4_) and sodium dihydrogen phosphate (NaH_2_PO_4_) (purchased from SRL chemicals, India) which was used as an electrolyte solution. Distilled water was obtained from Millipore ultrapure water system (18.2 MΩ.cm @ 25 ± 2 °C). During electrochemical studies, dissolved oxygen present in the electrolyte was removed by purging with nitrogen gas.

### 2.2. Instruments 

The functional groups of PEDOT:SCX/MXene were analysed by FT-IR via attenuated total reflection (ATR) mode by SHIMADZU, IRTRACER 100. For FT-IR analysis, the material was coated on a silicon wafer and dried completely. The microstructures and morphological features of PEDOT:SCX/MXene were characterized by a HR-SEM (Thermosceintific Apreo S). X-ray diffraction pattern (XRD) of MXene was analysed by XRD-PAN analytical Xpert Pro. Raman spectrum was recorded with a 532 nm excitation laser (LabRAM HR evolution, Horiba) connected to an Olympus imaging microscope with Labspec6 Raman software. X-ray photoelectron spectroscopy (XPS) measurement was performed using a PHI 5802 dual-console system. Electrochemical measurements (cyclic voltammetry and amperometry) were performed with an electrochemical workstation (CHI-760E, CH Instrument, Austin, TX, USA). A conventional three-electrode system was used with Ag/AgCl (3 M KCl) and platinum wire as the reference and counter electrodes. Glassy carbon electrode (GCE) (3 mm) modified with PEDOT:SCX/MXene/GOX film was used as the working electrode.

### 2.3. Synthesis of MXene Powder

**Figure 1:**
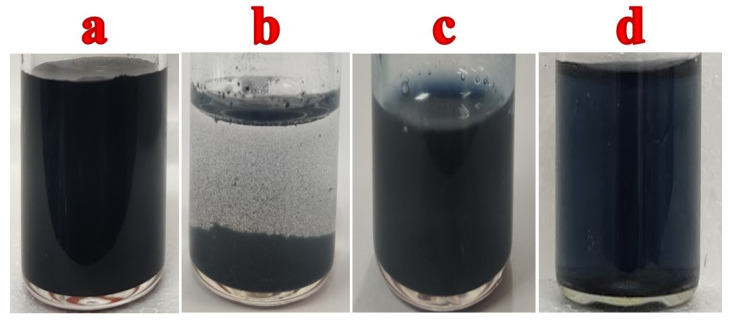
(**a**) MXene dispersion in water, (**b**) MXene/water dispersion after few hours, (**c**) PEDOT:SCX solution and (**d**) PEDOT:SCX/MXene dispersion in aqueous medium.

The Ti_3_C_2_T_x_ (MXene) was synthesized by using a high-temperature furnace under a nitrogen atmosphere. C, Al and Ti powders were mixed at a ratio of 3:1:2 and ball-milled for 12 h at room temperature. It resulted in black powder which was subjected to heating at 1100 °C for 2 h under a nitrogen atmosphere in a tubular furnace. The temperature of the furnace was increased at a rate of 5 °C min^−1^. Finally, it was cooled down to room temperature and the obtained product was etched with 40% HF under constant magnetic stirring at 1000 rpm for 24 h. Finally, the etched material was centrifuged and washed several times with distilled water to neutralize its pH and dried at 70 °C in a hot air oven to obtain MXene powder [40]. MXene dispersion was also prepared in an aqueous solution at a concentration of 0.1 mg/mL (Figure 1a). However, due to the poor dispersibility in water, MXene sheets were settled after a few hours (Figure 1b).

### 2.4. Polymerisation of EDOT

The chemical oxidative polymerization method was adapted to synthesis PEDOT [78]. In brief, 1 M FeCl_3_ (20 mL) was stirred using a magnetic bar at 800 rpm. Then, 0.51 mL of EDOT/ethanol (5 mL) was taken along with 1 mL of SCX (1 µM) solution. After that, EDOT/SCX mixture was added dropwise into the FeCl_3_ solution and kept for stirring up to 16 h. Finally, the obtained product was washed with water and ethanol to remove the unreacted substances and kept in a hot air oven for drying at 50 °C. The bluish-black colour PEDOT:SCX powder was obtained as shown in Figure S1. The PEDOT:SCX dispersion (0.1 mg/mL) was also prepared for electrode modification to study the electrochemical properties (Figure 1c). For the GOX immobilization, 1% chitosan solution and 1.5 mL of GOX was dissolved in 0.1 M PBS (pH 7.4). This mixture was bath sonicated for a few min and 5 μL of the (0.5 mg/mL) GOX solution was drop-casted on PEDOT:SCX/GCE and dried at ambient temperature.

### 2.5. Biosensor Preparation

**Scheme 1:**
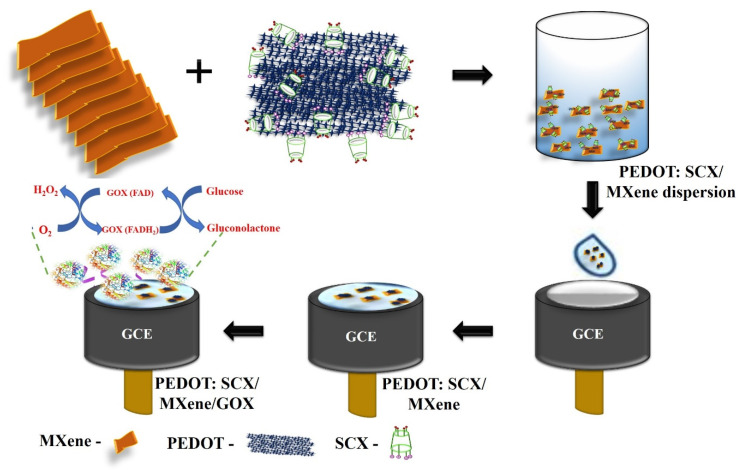
Schematic representation of electrode modification to prepare the glucose biosensor.

Initially, GCE was polished using a polishing pad with the sequence of alumina slurry (Al_2_O_3_) with the particles size ranged from 1 μm, 0.3 μm, and 0.05 μm. After that, GCE was rinsed well with ultrapure Milli-Q water and allowed to dry at room temperature. PEDOT:SCX/MXene (0.1 mg/mL) dispersion was prepared in water by bath sonication for 15 min and followed by probe-sonication for 30 min (Figure 1d). From this dispersion, 10 μL was drop-casted onto the GCE surface and dried at 50 °C. In parallel, 0.5 mL of 1% chitosan solution (binder) was added to 1.5 mL of GOX dissolved in 0.1 M PBS (pH 7.4). This mixture was bath sonicated for a few min and 5 μL of the GOX (0.5 mg/mL) solution was drop-casted on PEDOT:SCX/MXene/GCE and dried at 4 °C for few hours (Scheme 1). When the electrode was not in use, it was stored in the refrigerator with 0.1 M PBS (pH 7.4).

## 3. Results and Discussion

### 3.1. FT-IR Analysis

**Figure 2:**
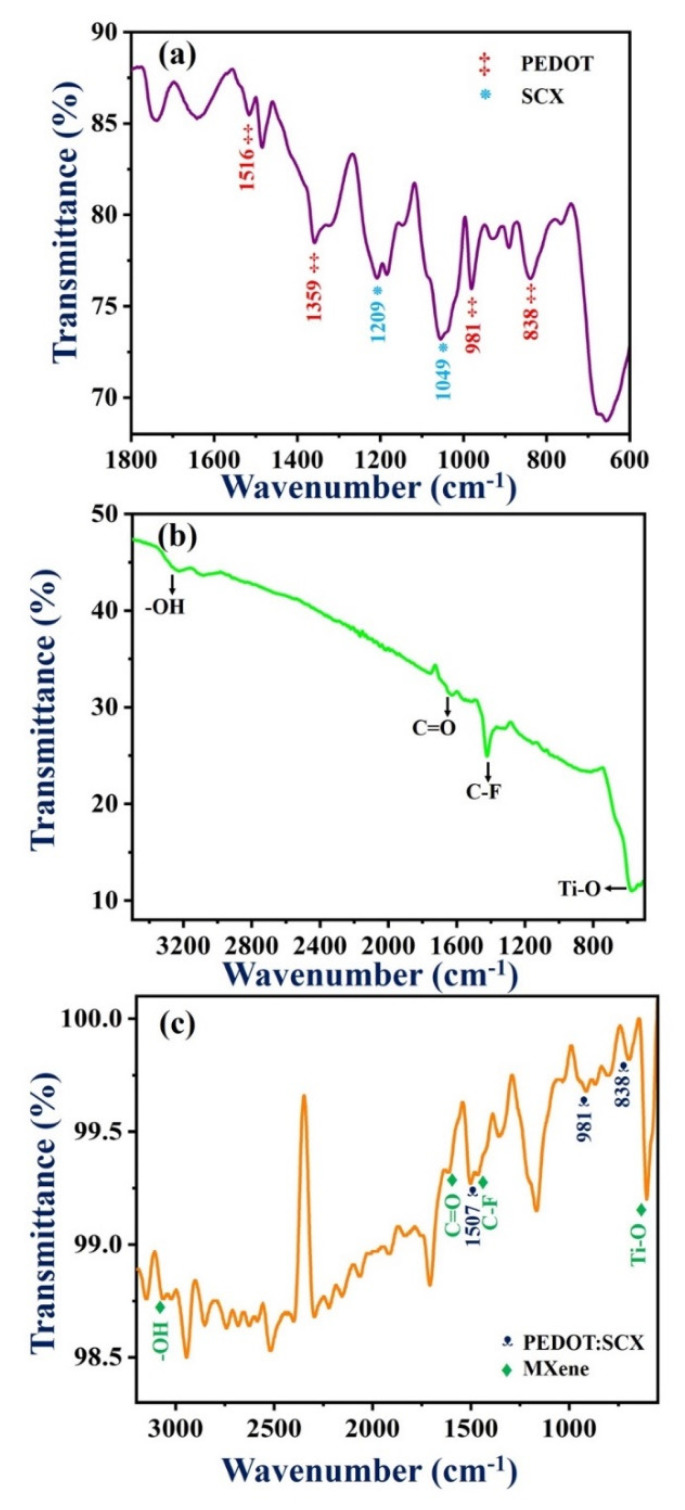
FT-IR spectra of (**a**) PEDOT:SCX, (**b**) MXene and (**c**) PEDOT:SCX/MXene.

Figure 2a shows the FT-IR characteristic peaks of PEDOT:SCX at 1541, 1516, and 1359 cm^−1^ which were attributed to the quinoidal structure and the stretching vibrations of the thiophene ring of C=C and C-C. The C-O-C bending vibrations in the dioxy-ethyl group were observed at 1184 and 1144 cm^−1^. The other IR bands found at 981, 931, 838, and 678 cm^−1^ were assigned to the stretching vibrations of C-S-C in the thiophene ring. The peak at 1037 cm^−1^ was observed due to the absorption of the –SO_3_H group which confirmed that the obtained PEDOT was doped with SCX [79]. The IR band of SCX was found at 1209 cm^−1^ indicated the splitting of the S=O group into symmetric and asymmetric stretching vibrations. The band at 1049 cm^−1^ denoted the asymmetric stretching vibration of the S-O group [80]. The redshift of the IR band corresponds to C=C stretching vibration was observed at 1541 cm^−1^, because of the doping of the conducting polymer in which the p-doped state was known for its highly stabilized form by electron-donating ethylene deoxy group [81,82]. This peak shift was also compared with the PEDOT spectrum as shown in Figure S2. In Figure 2b, the IR bands at 3310, 1417 and 1690 cm^−1^ were corresponded to the O-H vibrations, C-F vibrations, and C=O stretching vibrations. The metal oxide (Ti-O) bond vibration was found at 590 cm^−1^. In the FT-IR spectrum of PEDOT:SCX/MXene (Figure 2c), IR bands corresponded to the C=C of the thiophene ring in the PEDOT:SCX was blue shifted to 1507 cm^−1^. The metal oxide (Ti-O) peak was also blue-shifted from 590 to 588 cm^−1^. Due to PEDOT:SCX/MXene composite formation, an increase in the energy difference was observed by the blue shift. FT-IR study successfully confirmed the formation of PEDOT:SCX/MXene composite.

### 3.2. Raman Spectrum

**Figure 3:**
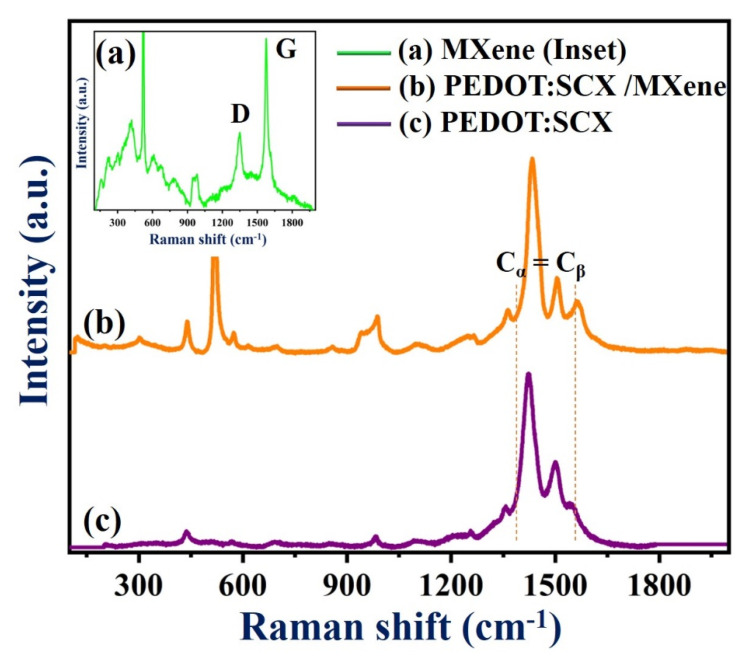
Raman spectra of (**a**) MXene, (**b**) PEDOT:SCX/MXene, and (**c**) PEDOT:SCX film.

Figure 3 (curve a) shows the Raman spectrum of MXene with the bands found at 220, 320, 617, and 688 cm^−1^. The Raman peaks at 617 and 680 cm^−1^ indicated the presence of Ti-C bond vibrations [40]. The E_g_ vibrations of TiO_2_ can be observed at 155 cm^−1^. Another two additional bands were observed at 1348 and 1571 cm^−1^ corresponded to the D and G bands of the graphitic carbon present in MXene. Figure 3 (curve b) shows the Raman spectrum (C_α_ = C_β_ stretching vibration) of PEDOT:SCX which exhibited bands at 1400 and 1500 cm^−1^ due to the doping on the PEDOT:SCX [81]. The changes in the Raman bands could be observed by comparing both spectra of PEDOT:SCX and PEDOT (Figure S3). Along with the stretching vibration, enhanced peaks at 1408 and 1556 cm^−1^ indicated the asymmetric stretching mode of C_α_ = C_β_ and the shift of stretching band was due to doping of the SCX in PEDOT [83]. The Raman band at 1364 cm^−1^ was observed due to the stretching of C_β_ = C_β’_ and another band located at 1263 cm^−1^ was due to C_α_ = C_α’_ inter-ring. The C-O-C deformation peak could be observed at 1106 cm^−1^ and the other peaks at 441, 573, and 986 cm^−1^ were ascribed to the deformation of the oxyethylene ring [81]. The symmetric C-S-C ring deformation of the polymer was also observed at 703 and 856 cm^−1^, respectively (Figure 3, curve c). 

In Figure 3c, the downshifted D and G bands can be observed at 1341 and 1504 cm^−1^. The G band intensity was lower than that of the D band and the I_D_/I_G_ ratio was found to be 0.56 which indicated that the defect was higher on the composite surface compared to pristine MXene. This may be due to the partially ordered crystal structure and defects present on the nanocomposite which also can provide favourable adsorption sites for enzyme immobilisation [84]. The deviations observed in the G band was due to the amorphization of graphite which indicated the presence of double bonds which resonates at a higher wavenumber. The crystallinity of Ti-C-Tx was also studied by XRD. As anticipated, the diffraction peaks (2θ values) at 35.9°, 41.8°, 60.5°, and 72.6° were equivalent to (111), (200), (220), and (311) planes of the Ti-C-Tx which denoted to the mixed phases of titanium carbide (Ti-C-Tx) (Figure S4). XRD pattern also exhibited bands at 2θ values of 25.5°, 37.7°, 48.3°, 54.3° (which were matched to TiO_2_), 52.5°, 57.3° and 77.0° (due to the presence of Al_2_O_3_), respectively [40].

### 3.3. XPS Analysis

**Figure 4:**
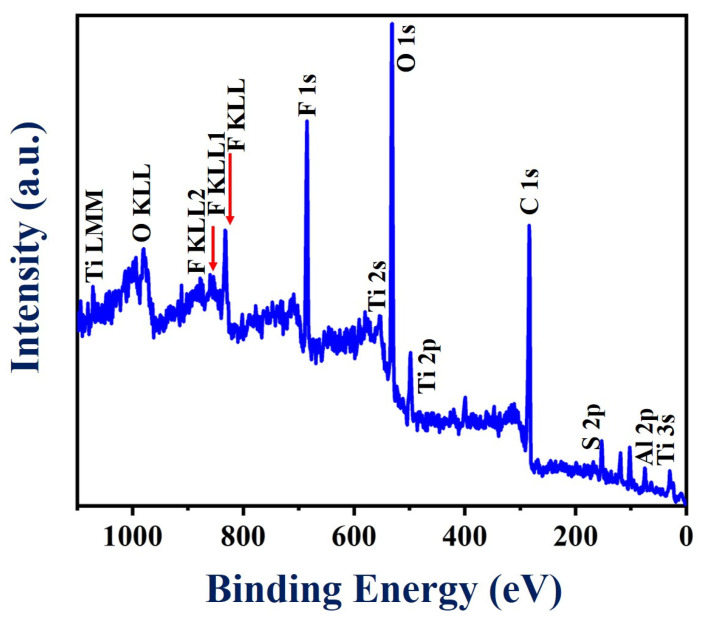
XPS survey spectrum of PEDOT:SCX/MXene powder.

To acquire more information about the surface chemistry of the PEDOT:SCX/MXene composite, XPS analysis was performed as shown in Figure 4. The survey spectrum showed the obvious signals of Ti, C, O, Al and F (Figure S5a–f). Specifically, various peaks were observed at binding energy values of 33, 120, 284, 453, 476, 530, 557, 593, 682, 734, 832 and 985 eV which were assigned to Al2s, C1s, Ti2p, O1s, Ti2s, F KLL, F1s, O KLL, Ti LMM and C KLL, respectively. In addition, XPS analysis also confirmed the presence of a small concentration of Al, which was due to the incomplete etching of Al from the Ti_3_AlC_2_ (MAX phase). (Figure S5f). All the related spectra corresponded to O1s, Ti2p and F1s were corrected with the C1s binding energy of 285 eV and deconvoluted using Gaussian fitting analysis. Accordingly, C1s spectra was (Figure S5a) split into three different types of peaks centred at 284.1, 286.8 and 289.9 eV corresponding to the C-C, C-O and C=O groups, respectively [85]. Moreover, the F1s peak (Figure S5b) was fitted into two peaks at 685.5 and 688.7 eV corresponding to Ti-F and Al-F functionalities [86]. Furthermore, Figure S5c showed three different types of O1s peaks with the binding energies of 531, 533.2 and 533.5 eV corresponding to Ti-O-Ti, C=O and -C-O functionalities, respectively [87]. The peak found at 459.1 eV in the Ti2p core level has been attributed to the presence of Ti(IV) in the TiO_2_ surface (Figure S5d). In addition, the peaks at 461.1 eV and 464.2 eV were assigned to Ti-C and Ti-F bonding in the composite material [86]. 

### 3.4. HR-SEM Analysis

**Figure 5:**
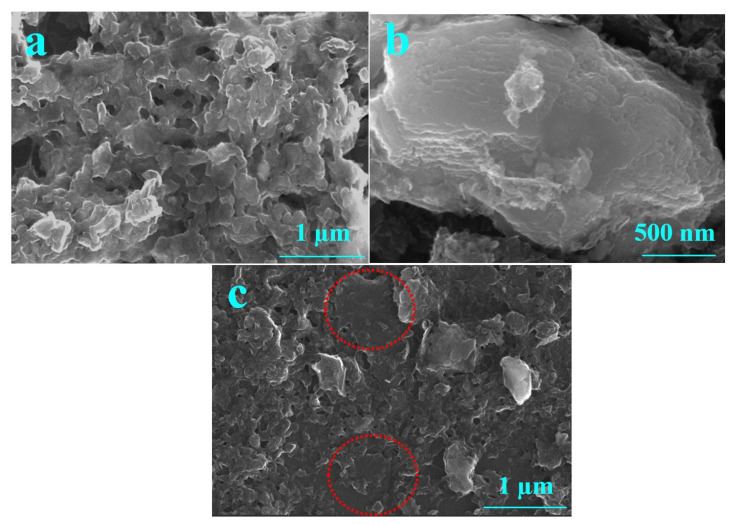
HR-SEM images of (**a**) PEDOT:SCX, (**b**) MXene and (**c**) PEDOT:SCX/MXene. The marked circle indicates the presence of MXene layer which was overlapped with PEDOT:SCX network.

Figure 5 shows the HR-SEM images of (a) PEDOT:SCX, (b) MXene and (c) PEDOT:SCX/MXene composite films. The PEDOT:SCX was found to have a network like sheet formations on the surface. A few-layered MXene flakes were observed for the synthesized material and the thickness of the MXene layer was found to be ~180 nm. The average size of a few-layer MXene was in the range of 400 to 500 nm. HR-SEM images of PEDOT:SCX/MXene confirmed the formation of nanocomposite with MXene and PEDOT:SCX. In addition, the Energy-dispersive X-ray spectroscopy (EDX) analysis has also confirmed the presence of C, S, Ti, F, and O in the nanocomposite (Figure S6).

### 3.5. Direct Electrochemistry of GOX

**Figure 6:**
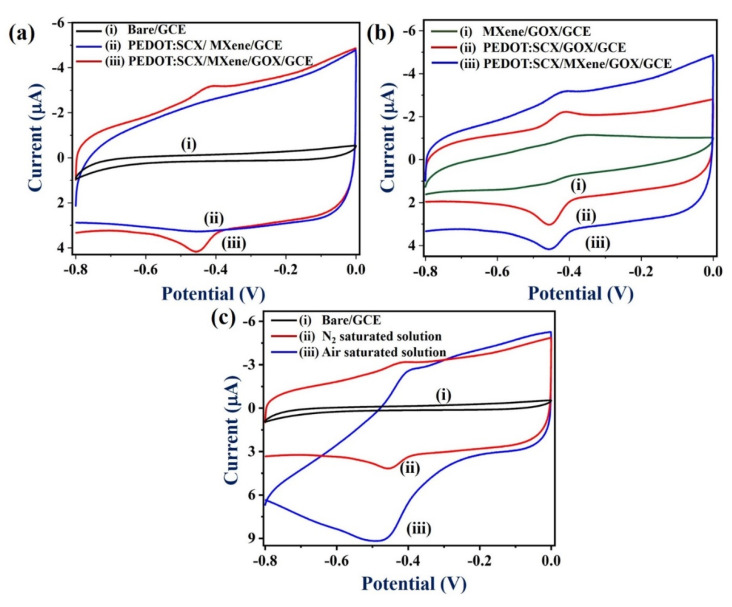
(**a**)**.** CVs of (i) bare GCE, (ii) PEDOT:SCX/MXene/GCE, and (iii) PEDOT: SCX/MXene/GOX/GCE, at a scan rate of 50 mV/s in 0.1 M PBS (pH 7.4) under N_2_ environment, (**b**) CVs of (i) MXene/GOX/GCE, (ii) PEDOT:SCX/GOX/GCE, and (iii) PEDOT:SCX/MXene/GOX/GCE in 0.1 M PBS (pH 7.4) at a scan rate of 50 mV/s under N_2_ environment. (**c**) CVs of (i) bare GCE, and (ii) PEDOT:SCX/MXene/GOX/GCE in the (ii) N_2_ saturated solution and (iii) in air saturated solution in 0.1 M PBS (pH 7.4) at a scan rate of 50 mV/s.

Next, the electrochemical properties of the PEDOT:SCX/MXene/GOX/GCE were studied by cyclic voltammetry (CV) in the potential range from 0 to −0.8 V at a scan rate of 50 mV/s in 0.1 M PBS (pH 7.4) under nitrogen (N_2_) atmosphere (Figure 6a,b) (curves iii). The anodic (E_pa_) and cathodic peak potentials (E_pc_) of GOX were found at − 0.42 V and −0.45 V, respectively. The formal potential (E° = (E_pa_ + E_pc_)/2) of GOX-FAD was estimated as −0.435 V due to the electron transfer between the PEDOT:SCX/MXene/GCE and GOX. This was in good agreement with other GOX immobilized electrodes [88]. The observed redox peak was due to the oxidation and reduction of the FADH_2_/FAD (electro-active centre) system present in the GOX enzyme (Equations (1) and (2)). Figure 6a, curve (i) represents CVs of the bare GCE in 0.1 M PBS at a scan rate of 50 mV/s under N_2_ environment which showed the non-faradaic current. In Figure 6a, curve (ii), PEDOT:SCX/MXene/GCE showed the capacitance behaviour where the electrostatic attraction takes place between the positively charged PEDOT:SCX and negatively charged MXene due to the surface functional groups. 

As shown in Figure 6b (curve i), the MXene/GOX/GCE did not show any redox activity of GOX which indicated the poor activity of GOX. It may be due to the poor immobilization of GOX on MXene/GCE due to electrostatic repulsion between the negatively charged MXene and negatively charged GOX. Therefore, the redox peak of GOX could not be observed. Figure 6b (curve iii) shows the CV curves of PEDOT:SCX/GOX/GCE where a well-defined redox peak was observed due to the immobilised GOX on the PEDOT:SCX modified electrode. The formal potential of PEDOT:SCX /GOX/GCE was found at –0.41 V. Notably, higher redox peak currents of GOX was observed on PEDOT:SCX/MXene/GOX/GCE which indicated that MXene/chitosan had helped in the process of GOX immobilization and provided good biocompatibility (Figure 6b, curves ii and iii). Chitosan consists of free amino and hydroxyl groups which can help to provide affinity between the materials (PEDOT:SCX/MXene and GOX) [89]. For example, the active amino groups in the chitosan backbone can provide active sites for side group attachment during bio-fabrication. The electrostatic attraction between the positively charged chitosan and negatively charged GOX might have also helped in the attachment of GOX in the composite film [90].

(1) $$\mathrm{GOX}\;(\mathrm{FAD})+2e^{-}+2H^{+}\;↔\;\mathrm{GOX}\;({\mathrm{FADH}}_{2})$$

GOX-FADH_2_ + O_2_→ GOX-FAD + H_2_O_2_ (2)

H_2_O_2_ → O_2_ +2e^−^ + 2H ^+^ (3)

Figure 6c shows the electrochemical activity of PEDOT:SCX/MXene/GOX/GCE in the presence of dissolved oxygen in PBS. For comparison, the CV response of the biosensor was also recorded in 0.1 M PBS (pH 7.4) (under N_2_ saturated) at a scan rate of 50 mVs^−1^ (Figure 6c, curve ii). In the air-saturated solution, PEDOT:SCX/MXene/GOX/GCE biosensor exhibited a sharp increase in the cathodic peak current with the significant decrease in the anodic peak current of GOX which was possibly related to the enhanced oxygen reduction reaction (ORR) on PEDOT:SCX/MXene/GOX/GCE (Figure 6c, curve iii). For control studies, CVs of the bare GCE were also recorded in 0.1 M PBS (pH 7.4) which showed no redox peak of GOX and poor ORR catalytic activity (Figure 6c, curve i) [91].

### 3.6. Effect of Scan Rate

**Figure 7:**
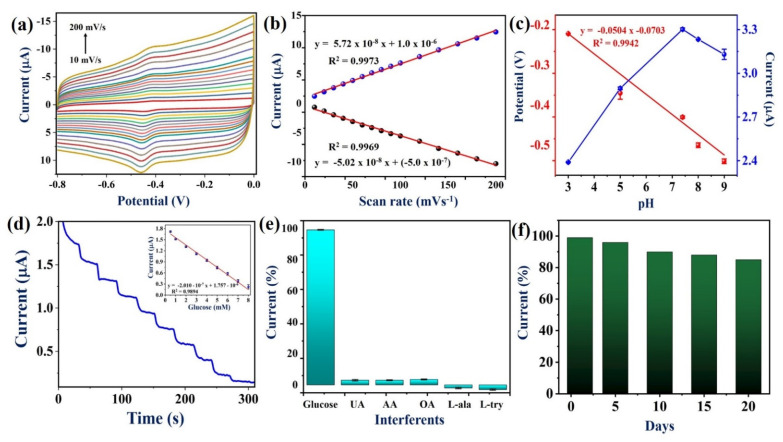
(**a**) CVs of effect of scan rates from 10 to 200 mV/s at PEDOT:SCX/MXene/GOX/GCE biosensor in 0.1 M PBS (pH 7.4) under N_2_ environment, (**b**) A linear plot of redox peak currents of GOX (I_pa_ and I_pc_) vs. scan rates, (**c**) The optimization of pH for PEDOT:SCX/MXene/GOX/GCE electrode in different pH (3, 5, 7.4, 8 and 9) buffer solutions, the linear plot for the modified electrode vs. pH, and I_pc_ vs. pH, (**d**) Amperometric response of PEDOT:SCX/MXene/GOX/GCE for continuous injection of glucose from 0.5 to 8 mM in 0.1 M PBS (pH 7.4) at an applied potential of −0.435 V. PBS was stirred at a rate of 950 rpm, (inset). A linear plot was made by using the reduction current vs. concentration of glucose (each datapoint is the mean of three measurements, *n* = 3), (**e**) Bar diagram represents the percentage changes in current response for PEDOT:SCX/MXene/GOX/GCE containing 1 mM glucose along with 1 mM of UA, AA, OA, L-ala and L-try. (**f**) Histogram of PEDOT:SCX/MXene/GOX/GCE’s stability analysis from 0 to 20th days of storage time.

The effect of scan rate on redox peak currents of PEDOT:SCX/MXene/GOX/GCE was also studied in 0.1 M PBS (N_2_ purged solution) by changing the scan rate from 10 to 200 mV/s (Figure 7a). A reversible GOX-FAD redox peak was found at the formal potential of −0.435 V vs. Ag/AgCl. When the scan rate increased, the redox peak currents also increased. From the plot of current vs. scan rate, it was found that the redox peak currents increased linearly with the correlation coefficient values of: (I_pa_) R^2^ = 0.9969 and (I_pc_) R^2^ = 0.9973 (Figure 7b). Therefore, the GOX-FAD redox reaction was a surface-controlled process on PEDOT:SCX/MXene/GOX/GCE (Equation (1)).

### 3.7. Effect of pH

The influence of pH on the PEDOT:SCX/MXene/GOX biosensor was also studied by CV. The GOX redox peak was pH dependent, and the peak potential was negatively shifted with the increase in pH from 3–9. As shown in Figure 7c, the relationship between the pH and redox potential of the GOX resulted in a slope of −50 mV/pH which was nearly equal to −59 mV/pH for the Nernstian response of the GOX redox reaction involved with the equal number of protons and electrons. At lower pH, the biosensor response for the H_2_O_2_ reduction was negligible. In this study, pH 7.4 was found to be the optimum pH for electrochemical sensing of glucose.

### 3.8. Amperometric Study of PEDOT:SCX/MXene/GOX/GCE for Glucose Sensing

Amperometric response of PEDOT:SCX/MXene/GOX/GCE was recorded for the sensing of glucose at an applied potential of −0.43 V under constant stirring at 950 rpm. Aliquots of glucose solutions were injected at a consistent interval of 30 s into uninterruptedly stirred air saturated PBS (pH 7.4). A well-defined and quick amperometric response was observed after each addition of glucose concentrations, and the response was stabilized within 15 s (Figure 7d). A linear calibration plot was drawn between the added glucose concentrations and the reduction currents (I_pc_) (Figure 7d inset) (Equation (4)).

I_pc_ = −2.010 × 10^−7^ x + 1.757 × 10^−6^ R^2^ = 0.9894 (4)

The LOD of glucose was estimated by using (Equation (5)) where σB is the standard deviation of the current for a blank sample and S is the slope of the calibration curve [92]. The LOD was calculated as 22.5 μM. The amperograms of glucose detection were recorded three times using a freshly fabricated PEDOT:SCX/MXene/GOX/GCE in order to calculate the standard deviation and error bars. The obtained results demonstrated that PEDOT:SCX/MXene/GOX/GCE biosensor was superior compared to some of the reported other enzymatic glucose sensors (Table 1).

LOD = 3σB/S(5)

### 3.9. Selectivity, Reproducibility and Repeatability Studies

The most important characteristic of the biosensor was the selectivity for the particular analyte in the presence of other interfering molecules. The selectivity of PEDOT:SCX/MXene/GOX/GCE against glucose was studied with several other biochemicals by CV. The selectivity of the biosensor was studied under the optimized condition with various interfering molecules, such as 1 mM of uric acid (UA), ascorbic acid (AA), oxalic acid (OA), L-alanine (L-ala) and L–tyrosine (L-try), which were added into the buffer solution with 1 mM of glucose (Figure 7e). Remarkably, there were no changes observed in the reduction currents upon adding the above-mentioned interferents. Furthermore, the repeatability study was also performed in the presence of 1 mM glucose in 0.1 M PBS using PEDOT:SCX/MXene/GOX/GCE biosensor for about three times, individually. Similarly, sensing of glucose was repeated in the time intervals of 1, 5, 10, and 20th days by using the same biosensor in 0.1 M PBS (Figure 7f). The relative standard deviation (RSD) of the current measurements was 2.1%. These data established that PEDOT:SCX/MXene/GOX/GCE biosensor had good stability with 87% of electrode response after storage for 20 days at 4 °C. This study exhibited that this biosensor had good reproducibility and repeatability when used as a glucose biosensor.

## 4. Real Sample Analysis

The real application of the biosensor was tested by detecting glucose concentration in fruit juice samples. The purchased fruit juice samples were shaken well and used without any pre-treatment. During the amperometry measurements, the specific concentration (2–4 mM) of glucose was spiked into the 0.1 M PBS with the fruit juice samples. The recovery percentages of spiked glucose concentrations were calculated to be 96 to 99% (Table 2). The obtained results indicated that the PEDOT:SCX/MXene/GOX biosensor can be used for the detection of glucose in the real samples. This experiment was also repeated three more times and the recovery percentages are shown in Table 2.

## 5. Conclusions

In this work, for the first time, the polymerization of EDOT was performed using SCX as a counter ion by the chemical oxidative method. Using PEDOT:SCX solution, the dispersibility of the MXene was achieved in water to obtain a stable PEDOT:SCX/MXene composite. The PEDOT:SCX/MXene composite was comprehensively characterized by using FT-IR, Raman, XRD, XPS and HR-SEM. The overall characterization results of PEDOT:SCX/MXene indicated that the functional groups of MXene helped to bind with the surface PEDOT:SCX film. Next, GOX was immobilized on the PEDOT:SCX/MXene/GCE with the help of chitosan as a binder. The selective application of PEDOT:SCX/MXene/GOX modified biosensor for glucose detection was demonstrated by using CV and amperometry techniques. The effect of pH on the biosensor response had been evaluated which revealed that the GOX redox reaction was involved with an equal number of electrons and protons. The analytical performance of the glucose biosensor was demonstrated by amperometry technique which showed that glucose can be detected from 0.5–8 mM with the LOD of 22.5 µM. Moreover, the PEDOT:SCX/MXene/GOX biosensor showed good reproducibility after continuous usage for 20 days. Finally, PEDOT:SCX/MXene/GOX electrode was applied to detect glucose in food samples. Thus, we believe that this novel PEDOT:SCX/MXene/GOX/GCE-based nanocomposite electrode will be helpful to fabricate biosensors for the detection of glucose in real-world samples.

## Figures and Tables

**Table 1:** Comparison of linear range and detection limit between the proposed sensor and other reported glucose sensors.

Electrode	Material Used for Modification	Linear Range in (mM)	Detection Limit (mM)	References
GCE	Graphene-chitosan-ZrO_2_	0.2–1.6	46	[93]
GCE	CEG	0.60–2.80	0.10	[22]
GCE	3DG-GOX	0.3–6	0.2	[94]
GCE	GOX/PPyAA/Au	1–18	0.05	[95]
PET substrate	Au/PEDOT-GOX	0–10	0.1	[96]
GCE	Co_3_O_4_@MCF-Chi-GOX	0–1.7	0.1077	[73]
GCE	PEDOT:SCX/MXene/GOX	0.5–8	0.0225	This work

CEG—chitosan encapsulated GOX, 3DG—3-dimensional graphene, PPyAA—pyrrole propylic acid, Au—gold, Co_3_O_4_@MCF—cobalt oxide supported mesoporous carbon framework, Chi—chitosan.

**Table 2:** The analysis of glucose in commercial samples using PEDOT:SCX/MXene/GOX/GCE biosensor.

S.No.	Samples	Added (mM)	Found (mM)	Recovery %	RSD ^a^ %
1	Fruit Juice	–	4	–	–
2	Fruit juice A	2	5.835	97.25	2.47
3	Fruit juice B	3	6.946	99.22	3.41
4	Fruit juice C	4	7.978	99.72	3.26

^a^ Mean value of three replicates.

## Data Availability

Data are available upon request.

## Supplementary Materials

The following supporting information can be downloaded at: https://www.mdpi.com/article/10.3390/mi13020304/s1, Figure S1. Visual images of PEDOT:SCX after washing off the impurities. Figure S2. FT-IR spectrum of PEDOT. Figure S3. Raman spectrum of PEDOT was recorded using 532 nm laser. Figure S4. XRD spectrum for MXene. Figure S5. XPS spectra of PEDOT:SCX/MXene, (a–e) high resolution spectra of C1s, F1s, O1s, Ti2p, S2p and Al2p regions, respectively. Figure S6. EDX spectrum of PEDOT:SCX/MXene nanocomposite.
