# Supplementary material for: Electrochemical Sensing of Glucose Using Glucose Oxidase/PEDOT:4-Sulfocalix [4]arene/MXene Composite Modified Electrode
Source: Micromachines (Basel). 2022 Feb 16;13(2):304. doi: 10.3390/mi13020304 (PMC8877456; doi:10.3390/mi13020304)
Supplement: Supplementary file 1 [file micromachines-13-00304-s001.zip › micromachines-1581248-Supplementary.pdf]

Supplementary Information

Electrochemical Sensing of Glucose Using Glucose Oxidase/PEDOT:4-Sulfocalix[4]Arene/MXene Composite Modified Electrode

Preethika Murugan[1,a], Jayshree Annamalai[2], Raji Atchudan[3,a], Mani Govindasamy[4], Deepak Nallaswamy[5], Dhanraj Ganapathy[5], Anatoly Reshetilov[6] and Ashok K. Sundramoorthy*[,1,5]

[1]Department of Chemistry, SRM Institute of Science and Technology, Kattankulathur-603 203, Tamil Nadu, India

[2]Department of Biotechnology, SRM Institute of Science and Technology, Kattankulathur, Tamil Nadu-603203, India

[3]School of Chemical Engineering, Yeungnam University, Gyeongsan 38541, Republic of Korea

[4]Department of Materials Engineering, Ming-Chi University of Technology, New Taipei City, Taiwan-243

[5]Department of Prosthodontics, Saveetha Dental College and Hospitals, Saveetha Institute of Medical and Technical Sciences, Poonamallee High Road, Velappanchavadi, Chennai, 600077, Tamil Nadu, India

[6]G.K. Skryabin Institute of Biochemistry and Physiology of Microorganisms, Pushchino Centre for Biological Research, Russian Academy of Sciences, 142290, Pushchino, Russian Federation

*Corresponding author

[a]These authors equally contributed.

Email: ashokkumars.sdc@saveetha.com ; ashok.sundramoorthy@gmail.com

Supplementary Figures

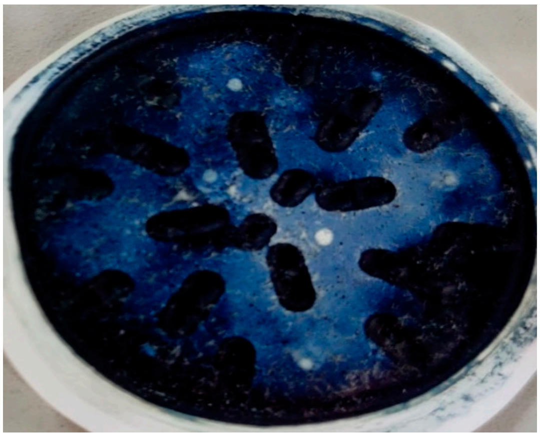

Figure S1. Visual images of PEDOT:SCX after washing off the impurities.

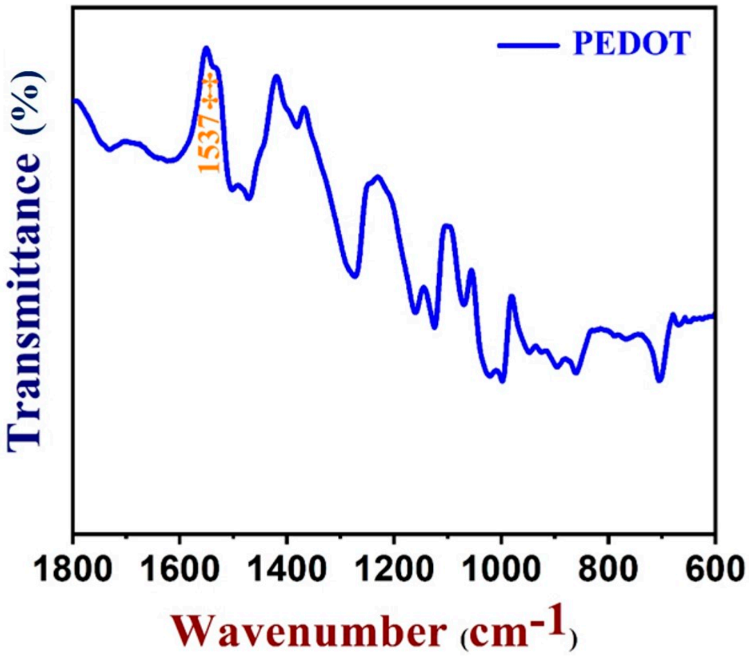

Figure S2. FT-IR spectrum of PEDOT.

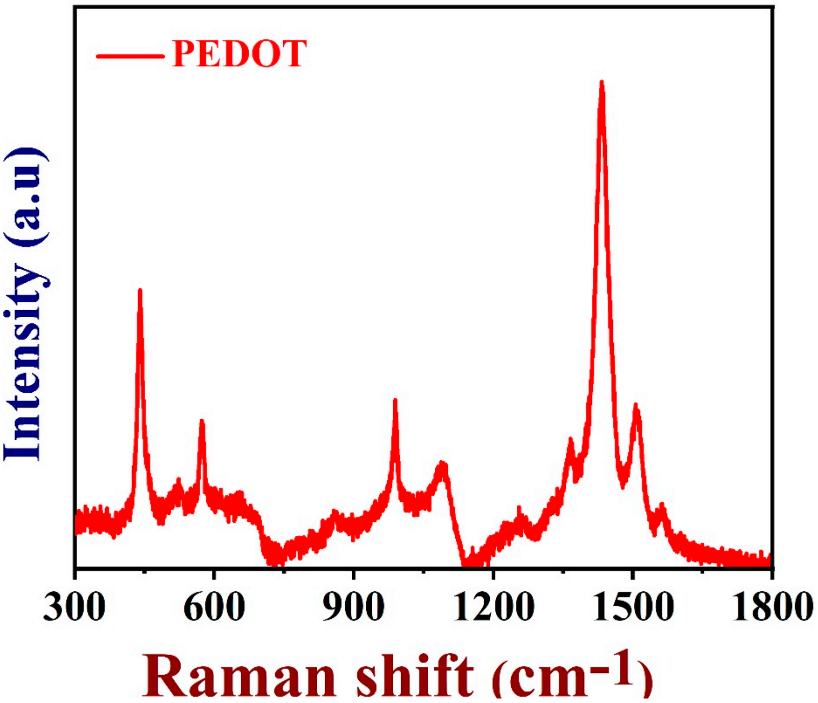

Figure S3. Raman spectrum of PEDOT was recorded using 532 nm laser.

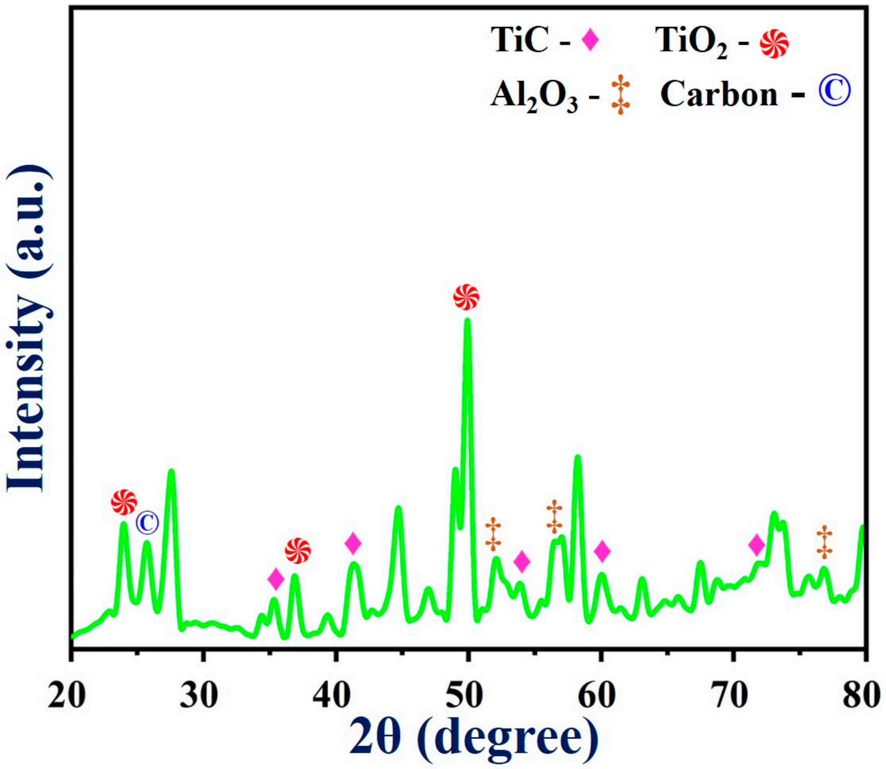

Figure S4. XRD spectrum for MXene.

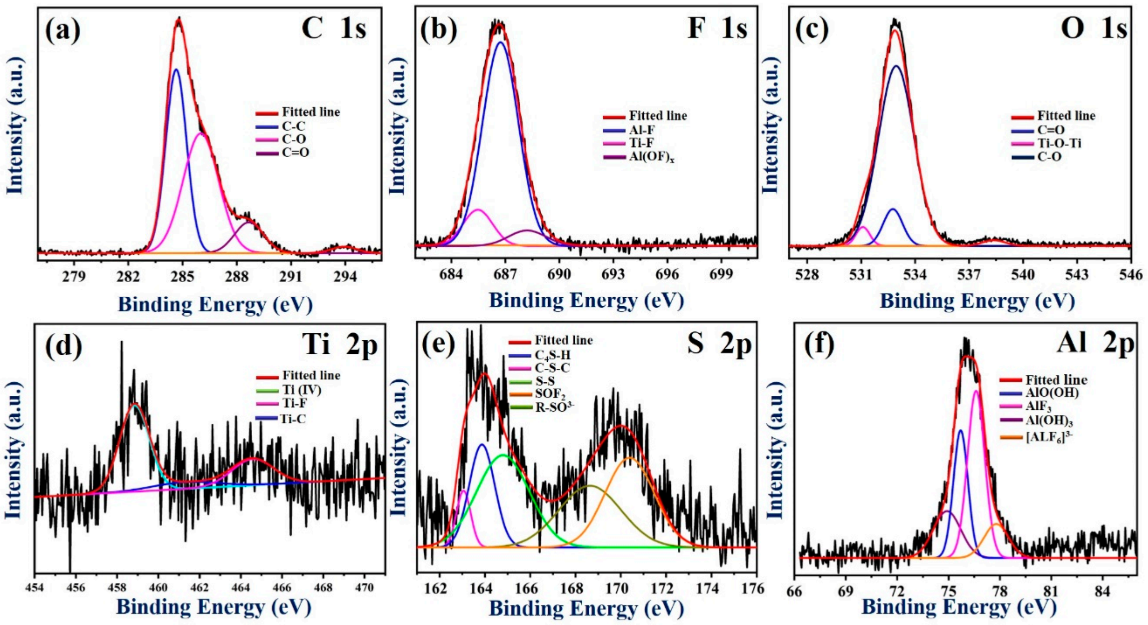

Figure S5. XPS spectra of PEDOT:SCX/MXene, **(a - e)** high resolution spectra of C1s, F1s, O1s, Ti2p, S2p and Al2p regions, respectively.

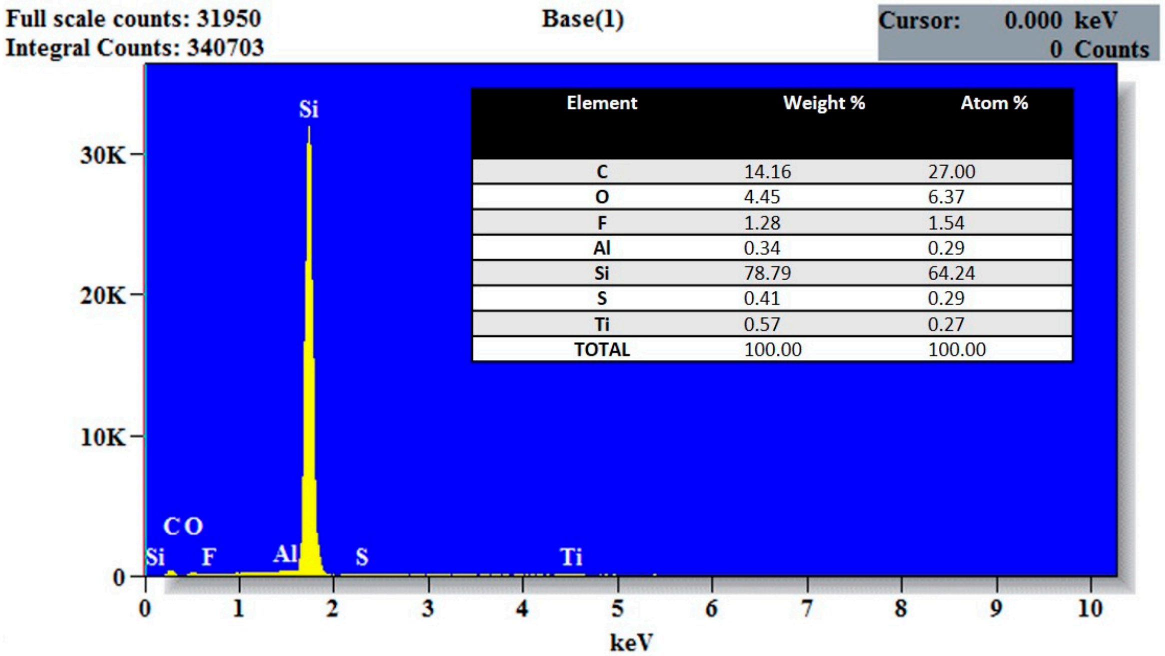

Element	Weight %	Atom %
C	14.16	27.00
O	4.45	6.37
F	1.28	1.54
Al	0.34	0.29
Si	78.79	64.24
S	0.41	0.29
Ti	0.57	0.27
TOTAL	100.00	100.00

Figure S6. EDX spectrum of PEDOT:SCX/MXene nanocomposite.
